# Supplementary material for: Simultaneous Recognition and Detection of Adenosine Phosphates by Machine Learning Analysis for Surface-Enhanced Raman Scattering Spectral Data
Source: Sensors (Basel). 2024 Oct 15;24(20):6648. doi: 10.3390/s24206648 (PMC11511347; doi:10.3390/s24206648)
Supplement: Supplementary file 1 [file sensors-24-06648-s001.zip › sensors-3228269-supplementary.pdf]

Simultaneous Recognition and Detection of Adenosine Phosphates by Machine Learning Analysis for Surface-Enhanced Raman Scattering Spectral Data

Contents

Table S1. Hyperparameters for each machine learning model.

Figure S1. SEM image of the gold plane (GP) with adsorbed nanoparticles.

Figure S2. FDTD simulation model details.

Figure S3. Comparison of reflection spectra between actual measurements (Exp.) and finite-difference time-domain simulations (Sim.).

Figure S4. Comparison of y-z cross-sectional enhanced electric field distributions at 785 nm.

Figure S5. Comparison between conventional Raman and SERS spectra of APs.

Figure S6. Results of the elbow method.

Figure S7. Confusion matrix of the machine learning models.

Table S1. Hyperparameters for each machine learning model.

Machine learning model	Hyperparameter	Value
Logistic regression (sklearn.linear_model.LogisticRegression)	-	-
Decision tree (sklearn.tree.DecisionTreeClassifier)	max_depth	3–10
	min_samples_split	2–10
	min_samples_leaf	1–10
k-nearest neighbor (sklearn.neighbors.KNeighborsClassifier)	n_neighbors	3–10
Linear discriminant analysis (sklearn.discriminant_analysis.LinearDiscriminantAnalysis)	n_components	1–6
Support vector machine (sklearn.svm.SVC)	C	1.0×10^{-5}–1.0×10^{5}
Random forest (sklearn.ensemble.RandomForestClassifier)	max_depth	3–10
	min_samples_split	2–10
	min_samples_leaf	1–10
Multilayer perceptron (sklearn.neural_network.MLPClassifier)	hidden_layer_sizes	(100–500, 0–200, 0–50)
	activation	logistic, tanh, relu
	solver	sgd, adam

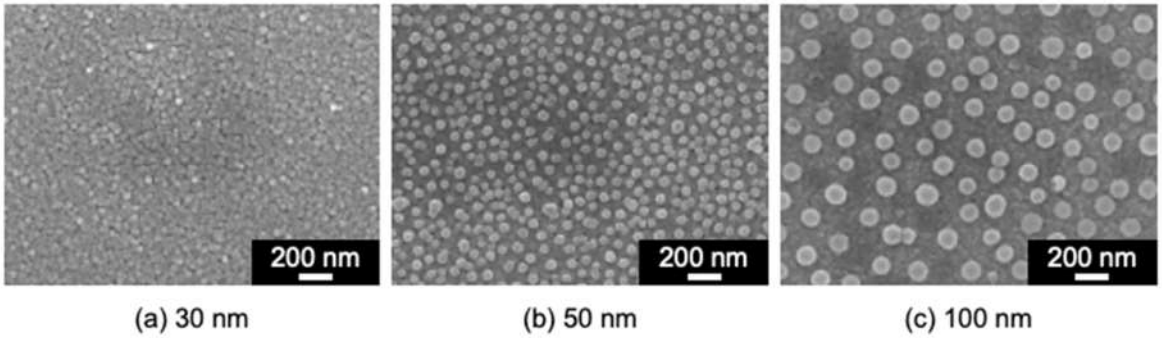

Figure S1. SEM image of gold plane (GP) with adsorbed nanoparticles.

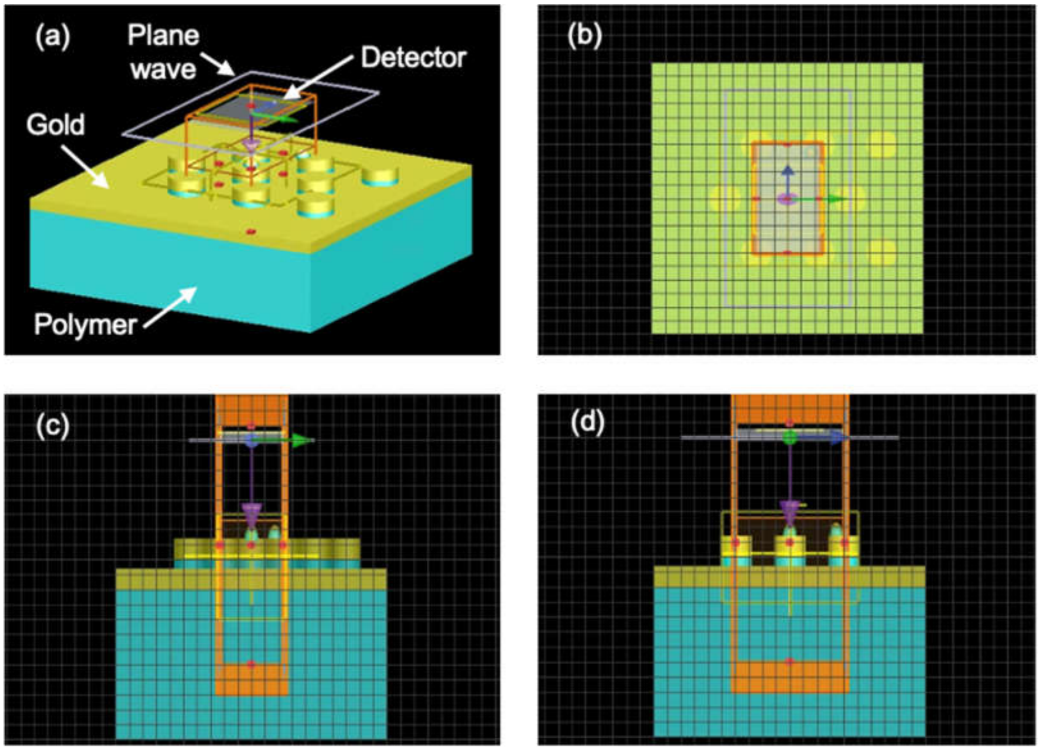

Figure S2. FDTD simulation model details. (a) 3D vision. (b) X-Y plane. (c) Z-X plane. (d) Y-Z plane.

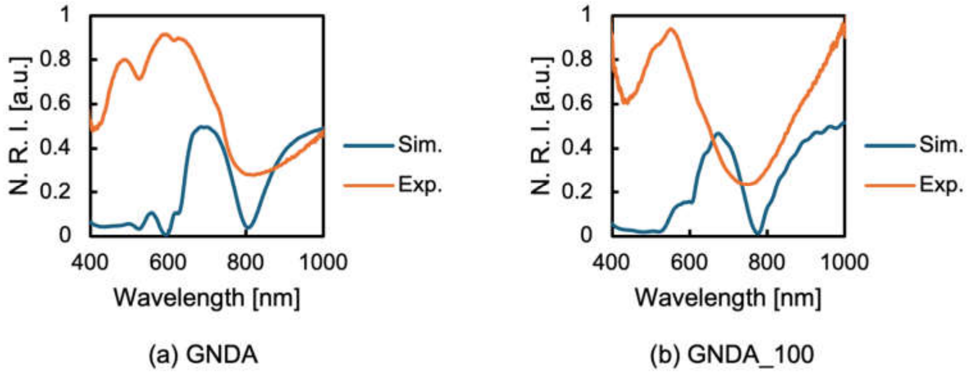

Figure S3. Comparison of reflection spectra between actual measurement (Exp.) and finite-difference time-domain simulation (Sim.). The vertical axis shows the normalized reflection intensity (N. R. I.).

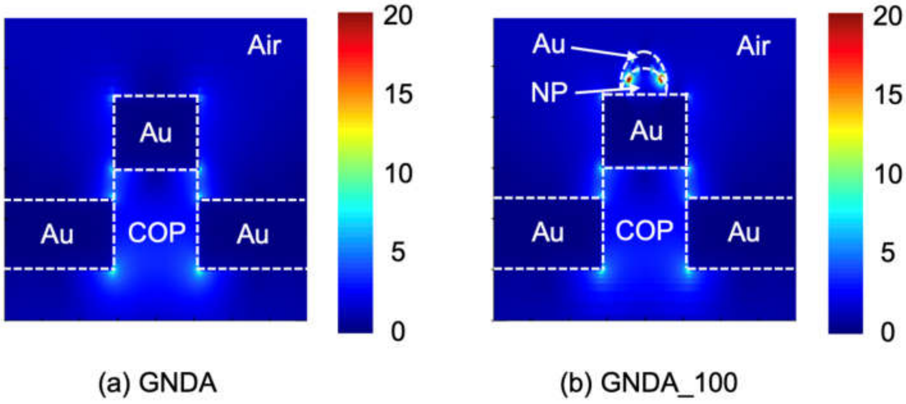

Figure S4. Comparison of y-z cross-sectional enhanced electric field distributions at 785 nm.

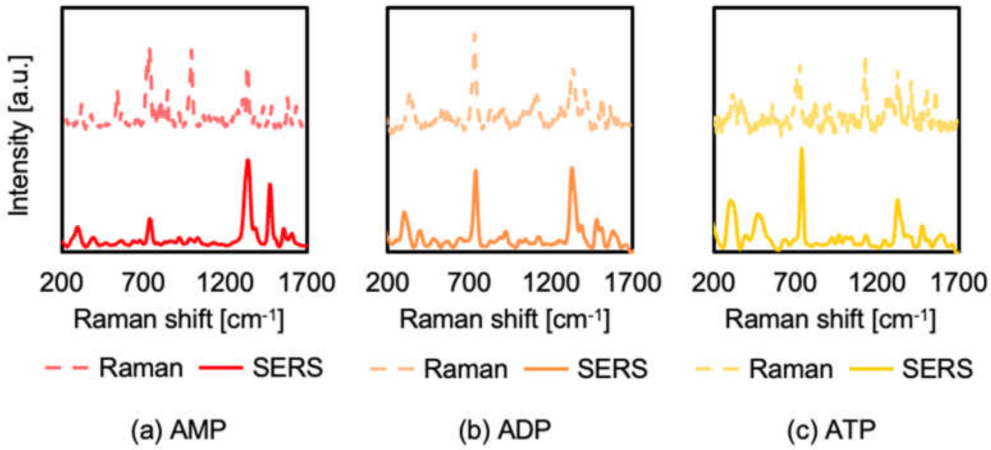

Figure S5. Comparison between conventional Raman and SERS spectra of APs.

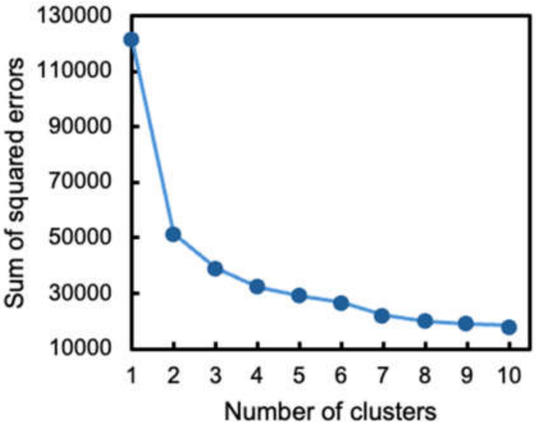

Figure S6. Results of the elbow method.

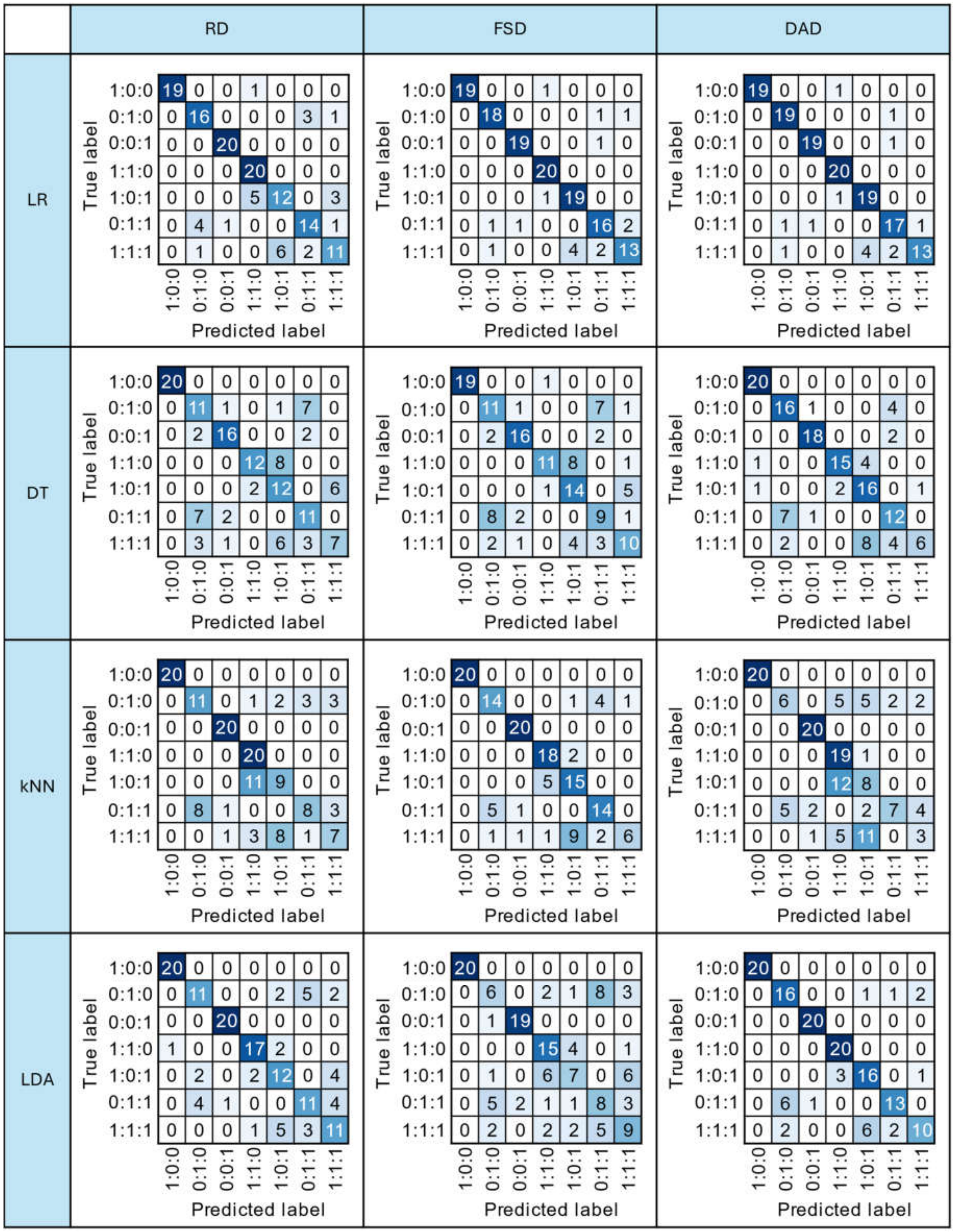
RD
FSD
DAD
LR
DT
kNN
LDA
True label
Predicted label
1:0:0
0:1:0
0:0:1
1:1:0
1:0:1
0:1:1
1:1:1

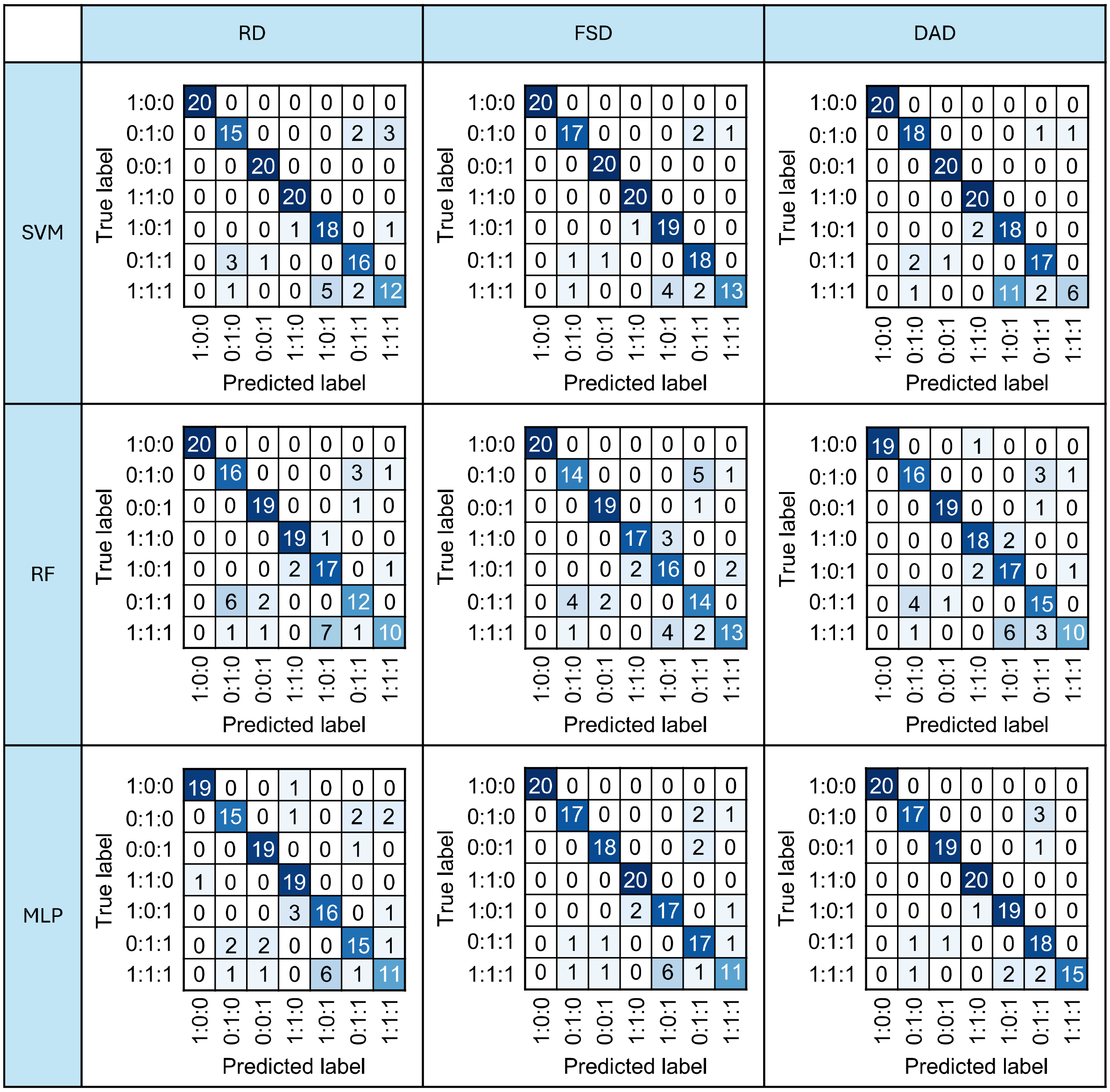

Figure S7. Confusion matrix of the machine learning models.
